# Evaluation of Performance of Welding Electrodes Containing Nano-Sized Rare Earth CeO_2_ Powder

**DOI:** 10.3390/ma19102103

**Published:** 2026-05-16

**Authors:** Aihua Wang, Xiuhua Shan, Jing Wang, Yun Peng, Lin Zhao, Yang Cao, Keping Zhai, Xianglei Kong

**Affiliations:** 1 Department of Mechanical Engineering, Hebei Petroleum University of Technology, Chengde 067000, China; hua23581@126.com; 2 Department of Petroleum Engineering, Hebei Petroleum University of Technology, Chengde 067000, China; zhai789978@163.com; 3 Industrial Technology Center, Hebei Petroleum University of Technology, Chengde 067000, China; jing237403@163.com; 4 China Iron & Steel Research Institute Group, Beijing 100081, China; 5 Department of Thermal Energy Engineering, Hebei Petroleum University of Technology, Chengde 067000, China; kong789978@163.com

**Keywords:** nanometer rare earth CeO_2_, iron powder welding rod, orthogonal experiment, process performance

## Abstract

**Highlights:**

- Nano CeO_2_ plays a significant role in weld formation, with a contribution rate of 76.92%.
- Iron powder has a significant impact on arc stability and slag removal, with contribution rates of 36.87% and 42.35%, respectively.
- Fluorite and iron powder have a significant impact on splashing, with a contribution rate of 43.24%.

**Abstract:**

Based on the E5018 zirconia–alkali low hydrogen iron powder electrode as the base composition, this paper incorporates nano-rare earth CeO_2_ powder into the coating. Using the orthogonal experimental method, a total of nine groups of experiments were established, each with four factors and three levels. These factors include (A) nano-rare earth CeO_2_ powder at levels of 1.1%, 1.3%, and 1.6%; (B) iron powder at levels of 30%, 35%, and 40%; (C) fluorite at levels of 6%, 8%, and 10%; and (D) zirconium quartz at levels of 5%, 7%, and 9%. The arc combustion stability of the welding rod is determined by an arc analyzer, and the formation and slag removal of the weld seam are evaluated by wide slope welding. Welding a spatter is evaluated by an observation method. The range and variance of the orthogonal experiment results were calculated, and the process performance was studied and analyzed. The results indicate that samples No. 1, 4, 5, 7, and 9 demonstrate superior performance in the welding process, specifically in terms of arc stability, weld formation, slag detachment, and spatter. The addition of nano-rare earth CeO_2_ powder has the most significant impact on weld formation, while iron powder, fluorite, and zirconium quartz have notable effects on arc stability, spatter, and slag detachment, respectively. The optimal combination of these four factors at three levels for optimal welding process performance is A2B1C2D3, with the recommended amounts being 1.3% of nano-CeO_2_ powder, 30% of the iron powders, 8% of fluorite, and 9% of zirconium quartz.

## 1. Introduction

Arc stability, slag detachability, and spatter rate are the primary technical indicators of the performance of basic electrodes. They not only reflect the process performance of basic electrodes during use and operation but also affect the formation of weld seams and the utilization rate of welding materials. Furthermore, they have a significant impact on the productivity, quality, and safety of welded structures [1].

Due to their low ionization energy, rare earth elements are easily ionized at high temperatures in the arc. This results in the production of charged particles, which contribute to stable arc combustion and enhance the arc initiation performance and conductivity characteristics. This makes the arc less prone to interruption and more stable, which is beneficial for enhancing arc stability [2,3,4]. Additionally, adding rare earth elements to the arc can reduce the spatter, promote the separation of slag and molten metal, and increase weld penetration, thereby improving weld formation [5]. Furthermore, the addition of fluorinated rare earths to submerged arc flux-cored wires adjusts the melting point and viscosity of slag, improves slag detachability, and reduces the diffusible hydrogen content of the deposited metal [6]. Thus, it has become crucial to include a suitable amount of rare earth additives in electrode coatings in order to improve the performance of electrodes [3,7] and the micro-structure of welds [8,9,10,11,12,13,14,15,16,17,18]. This has emerged as a significant area of research.

The transition of rare earths from droplets to the molten pool results in a significant loss, making it challenging to establish a direct correlation between them and the performance of the electrode process. Additionally, the nonlinear mathematical relationship between rare earth components further complicates this task, making it difficult to rely solely on traditional mathematical methods. Orthogonal experimentation is a tabular design method that is commonly used in exploratory research to study multiple factors and levels. It is particularly useful for studying factors such as electrode formulation design and corrosion testing. It is characterized by simple and feasible experimentation, tabular calculation, balanced distribution of points, fewer experimental trials, and strong representatives of the experimental results [4,7].

This study utilizes the advantages of high efficiency, speed, and economy in orthogonal experiments. Based on the typical E5018 alkaline electrode coating formula, nano-rare earth CeO_2_ powder is added to the coating. Using orthogonal experimental methods, a total of nine sets of experiments with four factors and three levels are established. The arc combustion stability of the electrode is measured by an arc analyzer, and the form-ability and slag removal of the weld seam are evaluated by wide slope welding. Welding spatter is evaluated by an observation method. The range and variance analysis of each indicator factor are completed, and the main and secondary order of each factor is determined by calculating the sum of squares and contribution rate. The contribution proportions of each process parameter to different quality indicators are quantified, providing clear priority guidance for subsequent process optimization and strong engineering application value. The results and process performance of the orthogonal experiment are also analyzed.

## 2. Materials and Methods

The experiment selected an H08A welding core. Specifications: diameter 3.2 mm, length 350 mm. The chemical composition of the welding core is shown in Table 1.

The base material used for the test is a 16Mn low alloy steel plate, the chemical composition is shown in Table 2 and the mechanical properties are shown in Table 3.

The welding rod preparation utilizes the TL-25A hydraulic welding rod coating machine, with a maximum working pressure of 25 kgf/mm^2^. The test pressure is set at 7–9 kgf/mm^2^ to ensure that the welding rod achieves a good appearance and appropriate coating strength.

The materials used in the experiment (welding core, plate, and electrode coating powder) are all produced in Baotou, China; The welding rod pressure coating machine is produced in Shijiazhuang, China.

**Figure 1:**
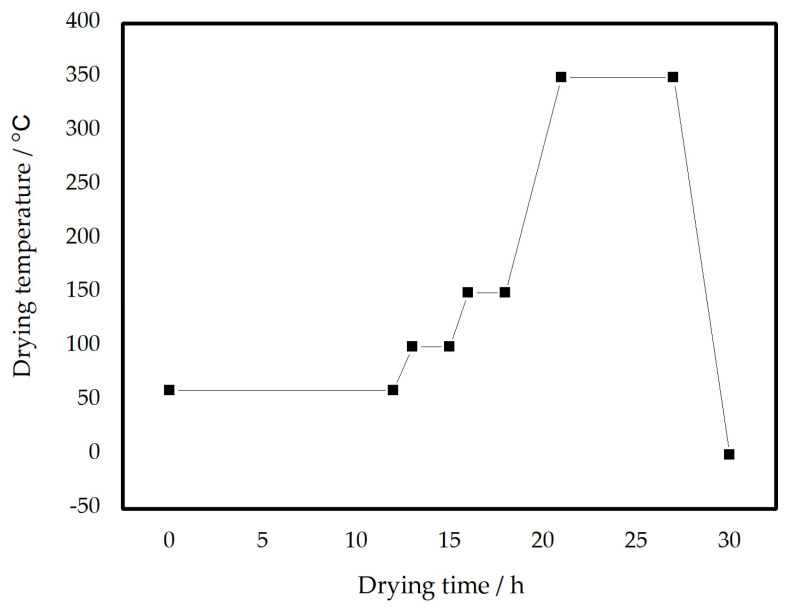
A warm-up ramp by heating ramp for the drying process.

The coating contains a high concentration of iron powder, at over 30%, which makes it susceptible to deformation and cracking during the drying process due to its thickness and weight. To prevent this, a specific drying process is used that involves carefully adjusting the temperature and gradually increasing it. The first step is to allow the coating to air dry for 24 h, followed by low-temperature drying for an additional 12 h. During this stage, the moisture is removed at 100 °C and 150 °C for an extended period. As the temperature rises from 150 °C to 350 °C, a controlled temperature adjustment method is used, increasing the temperature by 15 °C every 10 min to ensure a uniform temperature increase. The drying process is illustrated in Figure 1.

Measure the arc combustion stability of welding rods using the ANALYSATOR.

HANNOVER arc analyzer produced in Germany. The welding process parameters are current 150–160 A, voltage 24–30 V, and DC reverse connection. 

The weld seam formation and slag detachability were evaluated using the wide-slope welding method. The test procedure involved placing a test plate horizontally and holding the welding electrode at a 70° angle relative to the plate. A 20–25 mm wide weld seam was then deposited onto the plate and allowed to cool to room temperature before the slag was removed by tapping. The slag tapping was carried out by the same operator to ensure consistent tapping force throughout the process. The arc stability was evaluated using an arc analyzer and the criteria listed in Table 4. Visual observation was used to assess the weld seam formation, welding spatter, and slag detachability, with reference to the standards specified in Table 4.

## 3. Results

### 3.1. Coating Formula Design

The coating formula was developed based on the composition of the E5018 zirconia–alkali low-hydrogen iron powder electrode. It consists of 12 components, with the contents of eight components (titanium dioxide, zircon, potassium carbonate, quartz, medium carbon ferromanganese, low-grade ferrosilicon, ferrotitanium, and water glass) determined within the adjustable range of the E5018 reference formula. The range standard for the adjustment of alkaline low-hydrogen electrode coatings was also taken into consideration. In order to improve the slag removal of the alkaline electrode coating, a mixture of 50% micron-sized zirconium quartz (particle size 5–20 μm) and 50% nanometer-sized zirconium quartz (particle size 30–50 nm) was added to the coating. The contents of marble, fluorite, micron-sized iron powder, and nano-sized CeO_2_ powder play crucial roles in the welding electrode’s process performance and the mechanical properties of the weld metal. To determine the optimal contents of these four components, they were treated as variables in this study and their optimal contents were determined using orthogonal testing. The details are presented in Table 4. The range of the fluorite content was chosen to be between 6% and 10%, taking into consideration the principle that a content greater than 10% can make AC welding difficult and the fact that too little fluorite is not conducive to dehydrogenating the weld metal. ZrO_2_ was also treated as a variable, with a range of 5% to 9%, due to the poor slag detachability of alkaline welding electrodes. The iron powder content was set between 30% and 40%, considering that too much iron powder can lead to excessive dust emission during welding, while too little iron powder cannot improve the efficiency of the welding electrodes. To combine the advantages of nano-sized powders and rare earth oxides, this study selected nano-sized CeO_2_ powder (particle size 30–50 nm) and its content was determined by referring to the relevant literature. The marble content was set as the balance to ensure that the sum of all components was 100%. The components and contents of the coating formula are listed in Table 5.

### 3.2. Orthogonal Experimental Design of Electrode Coating Formula

Using the orthogonal experimental method, a four factor, three level orthogonal table L9 (3 × 4) was established, with a total of nine groups of experiments conducted. The table of experimental factors and levels is shown in Table 6, and the orthogonal experiments are presented in Table 7.

### 3.3. Welding Rod Process Performance Test Results

Based on the data presented in Table 6, a total of nine experiments were conducted using the orthogonal experimental method. The comprehensive balancing method was then utilized to analyze the results of the welding rod process performance, which are displayed in Table 8.

**Figure 2:**
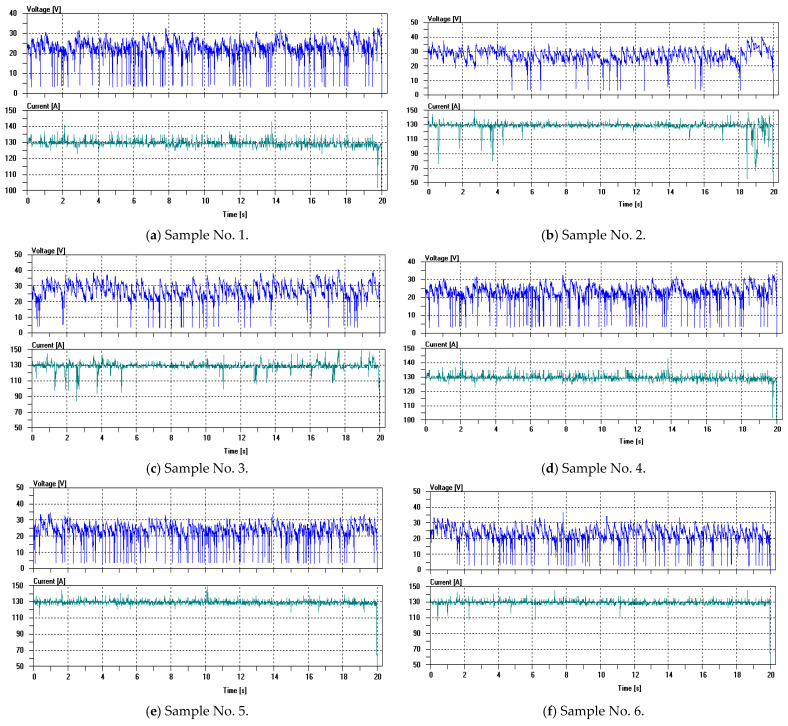

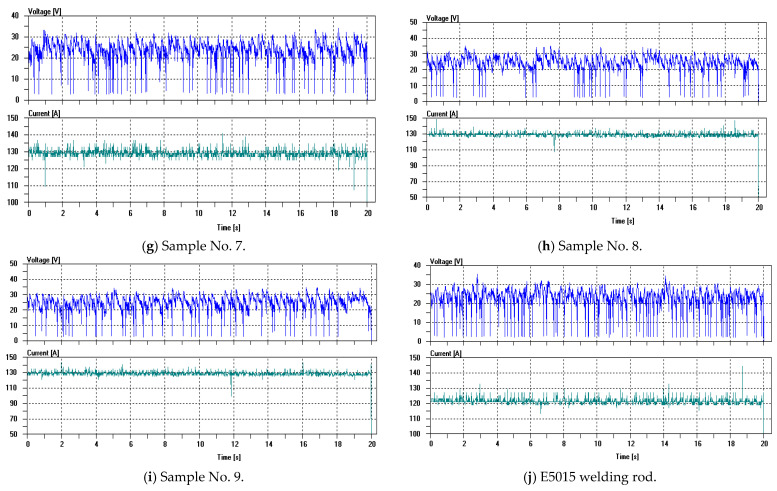
Voltage and current waveform diagram.

The voltage and current waveform diagrams for arc stability, which are among the experimental indicators, are shown in Figure 2:

To better assess the stability of the voltage and current during welds with nine different sets of electrodes, this study uses the waveform fluctuations of the voltage and current during welds with commercially available basic E5015 as a reference. This allows for a comparison of the arc stability between the different sets of electrodes. Figure 2j shows the voltage and current waveform diagram of E5015 used for reference and comparison. It can be seen that the voltage and current fluctuations of E5015 are smooth, with uniform voltage fluctuations and minimal current fluctuations. Each set of samples was tested for 20 consecutive seconds, starting from the time when the arc begins to burn stably. The results show that there were no interruptions in the arc during the combustion period for any of the nine sets of samples. By comparing the voltage and current waveform diagram of E5015 with the results from the nine sets of samples, it was found that samples No. 1, 4, 5, 7, and 9 exhibited smooth voltage and current fluctuations, indicating stable arc combustion. However, samples No. 6 and 8 showed occasional large fluctuations in the current or an unstable voltage during welding, and samples No. 2 and 3 exhibited relatively large fluctuations in the voltage and current, indicating unstable arc combustion.

### 3.4. Analysis of the Range and Variance of Welding Rod Process Performance

By calculating and analyzing Table 6, the range and variance analysis results of the welding rod process performance indicators were obtained, as shown in Table 9.

As can be seen from Table 9, among the four factors selected, nano-CeO_2_ powder has the greatest impact on weld formation among various indicators of process performance; iron powder, fluorite, and zirconium quart exhibit significant effects on the arc stability, spatter, and slag detachability, respectively.

**Figure 3:**
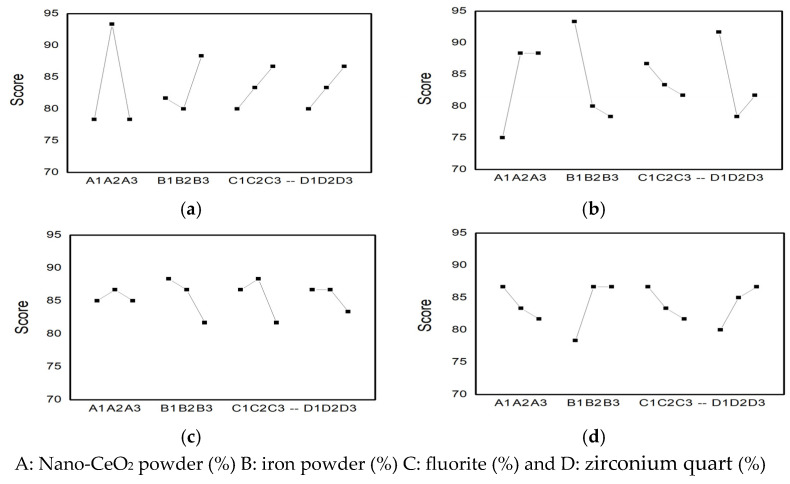
Four data and four factors of usability of electrode. (**a**) Relationship diagram between weld formation and four factors; (**b**) relationship diagram between arc stability and four factors; (**c**) relationship diagram between splash and four-factor; and (**d**) relationship diagram between slag removal and four factors.

Based on the results of the range analysis, a relationship diagram is presented in Figure 3, illustrating the impact of four factors on welding process performance (sub-indicators).

A1, A2, and A3 represent the nano-CeO_2_ powder contents of 1.1%, 1.3%, and 1.6%, respectively; B1, B2, and B3 represent the iron powder contents of 30%, 35%, and 40%, respectively; C1, C2, and C3 represent the fluorite contents of 6%, 8%, and 10%, respectively; and D1, D2, and D3 represent the zircon contents of 5%, 7%, and 9%, respectively.

According to Figure 3a and Table 9, the primary and secondary relationships of the factors affecting weld formation are as follows: nano-CeO_2_ powder → iron powder → fluorite ⟺ zirconium quartz. The most significant factor influencing weld formation is nano-CeO_2_ powder, with a contribution rate of 76.92%. The contribution rates of fluorite and zirconium quartz are equal at 6.2%.

The ideal combination for optimal levels is A2B3C3D3, which requires the following amounts: 1.3% nano-CeO_2_ powder, 40% iron powder, 10% fluorite, and 9% zirconium quart.

After examining the primary and secondary relationships of the four factors that affect weld formation, it is evident that the addition of nano-sized rare earth oxide CeO_2_ has the greatest impact. This is due to the fact that the appearance of weld formation is closely linked to the shape of the droplet, which is in turn influenced by the surface tension of the droplet. Therefore, reducing the surface tension of the droplet can greatly improve weld formation. In this study, the addition of nano-sized CeO_2_ powder results in the decomposition of the rare earth element Ce at high temperatures. As the rare earth element Ce is a surface-active element, it is able to adsorb onto the surface of the droplet under the high-temperature conditions of cathode spots and anode spots. This reduces the surface tension of the droplet, refines its shape, and ultimately improves weld formation [19].

According to Figure 3b and combined with Table 9, the primary and secondary relationships of factors affecting arc stability are as follows: iron powder → zirconium quart → nano-CeO_2_ powder → fluorite. The contribution rate of iron powder to the arc stability is 35.87%.

The optimal level combination is A2(A3)B1C1D1, with the following addition amounts: 1.3% or 1.6% of nano-CeO_2_ powder, 30% of the iron powders, 6% of fluorite, and 5% of zirconium quart.

From the voltage and current waveform diagram in Figure 2, it can be observed that during the arc combustion process, the fluctuations in the current and voltage are relatively stable. The stability of the current and voltage (excluding groups 2, 3, 6, and 8) is not significantly different from the E5018 voltage and current waveform diagram used for reference in Figure 2. This is mainly due to the role of iron powder in stabilizing the arc. At high arc temperatures, the iron in the coating evaporates into the arc space, increasing the concentration of the iron vapor. Gaseous iron atoms emit electrons under the high temperature and electric field of the arc, thus stabilizing the arc. Additionally, the rare earth element cerium, which is added to the coating, also plays a role in stabilizing the arc during the combustion process [20]. This is because rare earth elements have low ionization energies, as shown in Table 10 [21]. At high arc temperatures, they are more likely to ionize and release electrons. Furthermore, cerium group elements not only easily lose valence electrons from the s-shell, but can also lose an electron from the (n-2)f or (n-1)d shell. Therefore, they have a stronger ability to provide electrons. The addition of nano-CeO_2_ powder in this study is prone to decompose into cerium atoms at high temperatures. These rare earth cerium atoms have high chemical activity and can quickly decompose and ionize, thereby improving the conductivity of the arc, stabilizing the combustion process, and reducing the likelihood of arc interruption. As a result, the arc stabilization effect is more significant [22].

According to Figure 3c and combined with Table 9, the primary and secondary relationships affecting the splashing factors are as follows: fluorite ⟺ iron powder → zirconium quart → nano-CeO_2_. The influence of fluorite and iron powder on splashing is significant, with contribution rates of 43.24% for both.

The optimal level combination is A2B1C2D1 (D2), with the following addition amounts: 1.3% nano-CeO_2_ powder, 30% iron powder, 8% fluorite, and 5% or 7% zirconium quart.

From the primary and secondary relationships of the four factors affecting process performance indicators, it can be seen that fluorite has the greatest impact on the size of welding spatter. The main reason for this is that fluorite undergoes the following reaction at high temperatures:

CaF_2_ = Ca + 2F (1)

F + e → F^−^ (2)

The F element generated has a high electron affinity, causing it to adsorb electrons and form F^−^ ions in an arc atmosphere. However, due to the significantly greater mass of negative ions compared to electrons, they are not as effective in transferring charges. This results in reduced stability of the arc and increased spatter [23].

Figure 3d and Table 9 show the primary and secondary relationships between factors affecting slag removal, with iron powder, zirconium quartz, nano-CeO_2_ powder, and fluorite being the main contributors. The contributions of iron powder and zirconium quartz to slag removal are 42.35% and 27.07%, respectively.

The optimal combination is A1B2(B3)C1D3, with the following amounts added: 1.1% nano-CeO_2_ powder, 35% or 40% iron powder, 6% fluorite, and 9% zirconium quartz. The slag detachability of a weld is primarily related to the linear expansion coefficient, oxidizability, surface tension, and inter-facial tension of the molten slag.

The greater the difference in linear expansion coefficients between the slag and weld metal (14.5 × 10^−6^/°C for low carbon steel), the greater the difference in shrinkage between them during cooling, resulting in greater internal stress and better slag detachability.

To improve slag removal, efforts should be made to reduce the coefficient of linear expansion of the slag. As shown in Table 11, the coefficient of linear expansion of ZrO_2_ is relatively small. Therefore, in the development of the coating formula, some nano zirconium quartz was added to the electrode coating. Zirconium quartz decomposes into ZrO_2_ and SiO_2_ at high temperatures. During the cooling process, zirconium quartz undergoes phase transformation accompanied by volume expansion, and the phase transformation temperature of ZrO_2_ decreases with the decrease in the powder particle diameter. During the cooling process, large particles undergo transformation first, while small particles undergo transformation at lower temperatures. When the particles are small enough, ZrO_2_ can be stored at room temperature or even below room temperature. Therefore, adding some nanoscale zirconium quartz not only increases the expansion coefficient of the slag and the expansion coefficient of the weld metal during cooling, but also reduces the surface tension of the slag by retaining the untransformed zirconium dioxide [23], thereby better improving slag removal.

The addition of nano-CeO_2_ powder to the coating causes it to decompose into rare earth Ce atoms when exposed to high temperatures. These active rare earth Ce atoms possess highly reactive chemical properties and readily react with oxygen to produce stable rare earth oxides. This reduces the oxidizability of the slag and hinders the formation of an oxide film (FeO) on the surface of the weld metal. As a result, the weld metal maintains a metallic luster. The absence of an FeO oxide film on the surface of the weld metal also prevents spine compounds in the slag from adhering to it, resulting in excellent slag detachability [22].

The surface tension of slag and the inter-facial tension between slag and metal play a crucial role in the performance of welding electrodes. During the metallurgical reaction in the welding pool, the decomposition of CeO_2_ nanoparticles at high temperatures releases rare earth atoms that act as surface-active substances, reducing the surface tension of the weld metal [19]. Additionally, the small sizes of zirconium quart nanoparticles leads to an increase in the inter-facial tension difference between the slag and weld metal [23]. This creates a greater difference in internal stress during cooling and shrinkage, resulting in good slag detachability for the weld metal.

Based on the analysis above, the comprehensive equilibrium method of the orthogonal experiment was used to determine the optimal level combination of the four factors. This resulted in the combination A2B1C1D3 being identified as the optimal level combination for the process performance test. Although this combination was not included in the nine groups of experiments, it closely resembled the level factor combination A2B1C2D3 from the fourth group of experiments. Therefore, it can be approximated that the level factor combination from the fourth group of experiments is the optimal combination.

## 4. Conclusions

(1) The welding process performance of samples No. 1, 4, 5, 7, and 9 is relatively excellent in terms of arc stability, weld formation, slag removal, and spatter.
(2) Nano-CeO_2_ powder has the most significant influence on weld formation, while iron powder, fluorite, and zirconium quart have notable effects on arc stability, spatter, and slag detachment.
(3) The ideal combinations of the four factors (nano-CeO_2_ powder, iron powder, fluorite, and zirconium quart) at three levels for optimal process performance are as follows: weld formation → A2B3C3D3, arc stability → A2(A3)B1C1D1, spatter → A2B1C2D1(D2), and slag removal → A1B2(B3)C1D3.
(4) The optimal level combination is A2B1C2D3, with the following quantities: 1.3% nano-CeO_2_ powder, 30% iron powder, 8% fluorite, and 9% zirconium quart.

## Figures and Tables

**Table 1:** Chemical composition of H08A/wt%.

Element	C	Mn	Si	Cr	Ni	S	P
Content	≤0.01	0.30–0.55	≤0.20	≤0.030	≤0.30	≤0.030	≤0.030

**Table 2:** Composition and properties of base metal 16Mn/wt%.

Element	C	Si	Mn	S	P
Content	0.12~0.20	0.20~0.60	1.20~1.60	0.05	0.05

**Table 3:** Mechanical properties of base metal 16Mn.

Mechanical Performance	σ_s_/MPa	σ_b_/MPa	δ/%	A_kv_/J/cm^2^
Value	≥295	>490	>21	>59

**Table 4:** Evaluation criteria for experimental indicators.

Score	Evaluation criteria for weld formation
100–90	The weld seam is straight without obvious bending; the remaining height is 0–2 mm, uniform; width difference ≤ 1 mm; welding patterns are fine, uniform, and esthetically pleasing; no surface defects
89–80	The weld seam is relatively straight and slightly bent; the remaining height is 2–3 mm, basically uniform; width difference of 1–2 mm; welding pattern is relatively uniform; no cracks or lack of fusion; allow extremely small and tiny pores/pits (with a diameter of no more than 0.5 mm and no more than 2 points)
79–70	The weld seam has obvious bending; excess height of 3–4 mm or locally lower; width difference of 2–3 mm; welding pattern is coarse and uneven; allow a few tiny pores (with a diameter of ≤0.8 mm and ≤5 points) and slight undercutting (with a depth of ≤0.5 mm and a total length of ≤10% of the weld seams length)
69–60	Severe bending and sudden changes in width of the weld seam; excess height > 4 mm or <0 (concave); obvious biting edges, pores, slag inclusions, and pits, with a large quantity; no cracks, but defects exceed the standard
Below 60	There are fatal defects such as cracks, lack of fusion, dense pores, and large areas of slag inclusion; poor molding quality, unable to meet usage requirements
Score	Evaluation criteria for arc stability
100–90	Continuous and stable arc throughout the entire welding process, without arc interruption or extinguishing; voltage and current fluctuations are stable
89–80	The arc is basically stable, with slight arc blowing; voltage and current fluctuations are basically stable
79–70	The stability of the arc is average, with obvious arcs drifting or deviation; there are many fluctuations in voltage and current
69–60	The arc is clearly unstable, with severe arc oscillation or deviation; frequent voltage and current fluctuations
Below 60	The arc is extremely unstable, making it almost impossible to weld continuously, with frequent arc breaks and sticking of strips
Score	Evaluation criteria for splash
100–90	Basically no splashing or sporadic and extremely small splashes (with a diameter of less than 0.5 mm), ≤3 points/10 cm, easy to clean
89–80	A small amount of small splashes; particles with a diameter of 0.5–1.0 mm, ≤10 points/10 cm; distributed less than 10 mm from the weld seam, easy to clean
79–70	Moderate splashing; particles with a diameter of 1.0–1.5 mm, ≤20 points/10 cm; locally dense, 10–20 mm away from the weld, can be cleaned
69–60	A large amount of splashing; particle diameter > 1.5 mm, >20 points/10 cm; large area, dense, hard and difficult to clean, distance from weld seam > 20
Below 60	Serious and massive splashing; large particles (with a diameter greater than 2 mm), densely packed, and difficult to remove due to fusion with the base material; affects the surface quality of the base material
Score	Evaluation criteria for slag removal
100–90	Good automatics slag removal, with large areas falling off on their own after welding; 100% clean, with no slag or oxygen scale residue on the weld seam and both sides
89–80	Good slag removal, requiring slight tapping to detach; basic clean up; allow a very small amount of tiny slag points (less than 0.5 mm, ≤2 points/10 cm), easy to remove
79–70	Generally, slag removal requires strong tapping; a small amount of slag residue: diameter 0.5–1.0 mm, ≤5 points/10 cm; no large area of adhesive slag skin
69–60	Poor slag removal, hard and adhered slag skin, difficult to remove in large pieces; more slag residue: >1.0 mm, >5 points/10 cm; there are obvious slag blocks in the weld and groove
Below 60	Poor slag removal, slag and weld fusion; large scale slag coverage and uncleaned; serious impact on appearance and subsequent testing

**Table 5:** Composition and content of formula of electrode cover.

Ingredient	Content/wt%	Ingredient	Content/wt%
titanium dioxide	3	CeO_2_ nano-rare earth oxide	1.1~1.6
potassium carbonate	1	50% reduced iron powder + 50% micron iron powder	30~40
Quartz	6	fluorite	6~10
medium carbon	6	zirconium quart (50% micron + 50% nano)	5~9
low silicon ferrosilicon	3	(marble) margin	12~30
ferrotitanium	9	water glass	25 mL

**Table 6:** Table of experimental factors and levels/wt%.

Test Number	CeO_2_ Nano-Rare Earth Oxide (A)	Iron Powder (50% Reduced + 50% Micron) (B)	Fluorite (C)	Zirconium Quart (50% Micron + 50% Nano) (D)
1	1.1 (A_1_)	30 (B_1_)	6 (C_1_)	5 (D_1_)
2	1.3 (A_2_)	35 (B_2_)	8 (C_2_)	7 (D_2_)
3	1.6 (A_3_)	40 (B_3_)	10 (C_3_)	9 (D_3_)

**Table 7:** Table of orthogonal experimentation/wt%.

Test Number	CeO_2_ Nano-Rare Earth Oxide (A)	Iron Powder (50% Reduced + 50% Micron) (B)	Fluorite (C)	Zirconium Quart (50% Micron + 50% Nano) (D)	Margin (Marble)
1	1.1	30	6	5	30
2	1.1	35	8	7	20
3	1.1	40	10	9	12
4	1.3	30	8	9	24
5	1.3	35	10	5	21
6	1.3	40	6	7	18
7	1.6	30	10	7	23
8	1.6	35	6	9	20
9	1.6	40	8	5	17

**Table 8:** Process performance of electrode.

Test Number	A	B	C	D	Test Indicators and Score			
					Weld Formation	Arc Stability	Splash	Slag Removal
1	1.1	30	6	5	70	95	90	80
2	1.1	35	8	7	75	65	90	90
3	1.1	40	10	9	90	65	75	90
4	1.3	30	8	9	95	95	90	80
5	1.3	35	10	5	90	90	85	80
6	1.3	40	6	7	95	80	85	90
7	1.6	30	10	7	80	90	85	75
8	1.6	35	6	9	75	85	85	90
9	1.6	40	8	5	80	90	85	80

Note: The evaluation of the score size in the experimental indicators is shown in Table 4.

**Table 9:** Range analysis and variance analysis table of welding rod process performance indicators.

Indicator	Weld Formation				Arc Stability				Splash				Slag Removal			
Factor	A	B	C	D	A	B	C	D	A	B	C	D	A	B	C	D
K_1_	230	245	240	240	225	280	260	275	255	265	260	260	260	235	260	240
K_2_	280	240	250	250	265	240	250	235	260	260	265	260	250	260	250	255
K_3_	235	265	260	260	265	235	245	245	255	245	245	250	245	260	245	260
K_1_	78.33	81.67	80	80	75	93.33	86.67	91.67	85	88.33	86.67	86.67	86.67	78.33	86.67	80
K_2_	93.33	80	83.33	83.33	88.33	80	83.33	78.33	86.67	86.67	88.33	86.67	83.33	86.67	83.33	85
K_3_	78.33	88.33	86.67	86.67	88.33	78.33	81.67	81.67	85	81.67	81.67	83.33	81.67	86.67	81.67	86.67
R	15	8.33	6.67	6.67	13.33	15	5	13.34	1.67	6.66	6.66	3.34	5	8.34	5	6.67
MS	167.39	23.23	13.50	13.50	32.22	40.56	4.4	32.78	0.94	15.11	15.11	3.78	10.50	29.08	10.50	18.59
factor sorting	1	2	3	3	3	1	4	2	3	1	1	2	3	1	3	2
contribution rate/%	76.92	10.67	6.20	6.20	29.29	36.87	4.04	29.80	2.70	43.24	43.24	10.82	15.29	42.35	15.29	27.07

**Table 10:** First ionization energy of rare earth element/ev.

Y	La	Ce	Pr	Nd	Al	Mg	Ca
6.38	5.577	5.60	5.48	5.5	5.986	7.646	6.1

**Table 11:** Factor of line expand of oxide/k^−1^.

Oxide	Li_2_O	Na_2_O	MgO	K_2_O	BeO	CaO	SrO	BaO
α_i_	27(27)	39.5(41)	6.0	46.5(50)	4.5	13	16	20
**Oxide**	**B_2_O_3_**	**Al_2_O_3_**	**SiO_2_**	**ZrO_2_**	**TiO_2_**	**P_2_O_5_**	**ZnO**	**PbO**
α_i_	−5.0bis0.0	−3.0	0.5bis3.8	−6.0	−1.5bis + 3.0	14.0	5.0	6

## Data Availability

The original contributions presented in this study are included in the article. Further inquiries can be directed to the corresponding author.
